# Fractional anisotropy thresholding for deterministic tractography of the roots of the brachial plexus

**DOI:** 10.1038/s41598-020-79840-8

**Published:** 2021-01-08

**Authors:** Ryckie G. Wade, Irvin Teh, Gustav Andersson, Fang-Cheng Yeh, Mikael Wiberg, Grainne Bourke

**Affiliations:** 1 Academic Plastic Surgery Office, Department of Plastic and Reconstructive Surgery, Leeds General Infirmary, Leeds Teaching Hospitals Trust, Leeds, LS1 3EX UK; 2 Faculty of Medicine and Health Sciences, University of Leeds, Leeds, UK; 3 Leeds Institute for Cardiovascular and Metabolic Medicine, University of Leeds, Leeds, UK; 4 Department of Integrative Medical Biology, Faculty of Medicine, Umeå University, Umeå, Sweden; 5 Department of Surgical and Perioperative Science, Faculty of Medicine, Umeå University, Umeå, Sweden; 6 Wallenberg Centre for Molecular Medicine, Umeå University, Umeå, Sweden; 7 Department of Neurological Surgery, University of Pittsburgh, Pittsburgh, USA

**Keywords:** Peripheral nervous system, Preclinical research, Neurology

## Abstract

Diffusion tensor imaging (DTI) metrics, such as the fractional anisotropy (FA) and estimates of diffusivity are sensitive to the microstructure of peripheral nerves and may be displayed as tractograms. However, the ideal conditions for tractography of the roots of the brachial plexus are unclear, which represents the rationale for this study. Ten healthy adults were scanned using a Siemens Prisma (3T) and single-shot echo-planar imaging (b-value 0/1000 s/mm^2^, 64 directions, 2.5 mm^3^ with 4 averages; repeated in opposing phase encoding directions). Susceptibility correction and tractography were performed in DSI Studio by two independent raters. The effect of FA thresholding at increments of 0.01 (from 0.04 to 0.10) were tested. The mean FA varied between subjects by 2% (95% CI 1%, 3%). FA thresholds of 0.04, 0.05 and 0.06 all propagated 96% of tracts representing the roots; thresholding at 0.07 yielded 4% fewer tracts (*p* = 0.2), 0.08 yielded 11% fewer tracts (*p* = 0.008), 0.09 yielded 15% fewer tracts (*p* = 0.001) and 0.1 yielded 20% fewer tracts (*p* < 0.001). There was < 0.1% inter-rater variability in the measured FA and 99% agreement for tractography (κ = 0.92, *p* < 0.001). The fractional anisotropy thresholds required to generate tractograms of the roots of the brachial plexus appears to be lower than those used in the brain. We provide estimates of the probability of generating true tracts for each spinal nerve root of the brachial plexus, at different fractional anisotropy thresholds.

## Introduction

The structures most commonly affected in adults with a brachial plexus injury (BPI) are the spinal nerve roots^1^. Dedicated magnetic resonance imaging (MRI, including imaging in multiple planes with different sequences) is the best non-invasive test for diagnosing root injury although it misclassifies up to 25% of in-continuity nerve roots as avulsed^1^, meaning that many surgeons still undertake exploratory surgery. This shortfall in conventional imaging might be improved by diffusion tensor imaging (DTI), which is sensitive to changes in the microstructure of peripheral nerves (e.g. myelination, axon population, fibre organisation, etc.)^2^ through metrics such as the fractional anisotropy (FA), mean diffusivity (MD), axial diffusivity (AD) and radial diffusivity (RD). Further, DTI tractography may help clinicians to evaluate changes in the diffusivity and anisotropy throughout the length of the roots, which we speculate might help to identify areas of pathology or targets for surgery.

Previous studies have shown the feasibility of DTI tractography of the brachial plexus in adults^3–8^ and others have shown the reproducibility of DTI metrics without tractography^9,10^. Tagliafico^4^ and Vargas^3^ used different FA thresholds for tractography across patients, citing thresholds of 0.15 +/− 0.05 but it is unclear how and from where these values were measured, and what the +/− 0.05 represents. Tagliafico^4^ does not report the frequency of propagated tracts whilst Vargas^3^ propagated C5-T1 tracts in all healthy volunteers. Conversely, work from our group^8^ and Oudeman^5^ used fixed FA thresholds of 0.06 and 0.1, respectively. Oudeman^5^ reconstructed fibres representing the C5–C8 root in all cases, but in 52% of datasets, the 1^st^ thoracic root was not reconstructed. Similarly, we propagated tracts of the C5–C8 roots in 96% of cases, although the T1 root was only visualised in 54% of datasets. Neither Gasparotti^6^ or Su^7^ described the FA thresholds they used for tractography, with the former reconstructing all roots in all subjects and the latter only reporting on the C5-8 roots.

Before DTI can be used to supplement the assessment the roots of the brachial plexus in patients, numerous questions concerning the acquisition of diffusion data, correction of artefacts, modelling approaches and data analysis must be answered. One such issue is the FA threshold used for tractography. It is widely accepted that the FA threshold has a significant effect on white matter tractography; however, the effect of different FA thresholds on tractograms of the brachial plexus and the extraction of tract-related metrics remains unknown, which forms the rationale for this study.

## Methods

This single-centre study was designed and reported in accordance with the STARD guidance, taking into account the domains of the QUADAS-2 and PRISMA-DTA tools. Approval was gained from the National Research and Ethics Service of the United Kingdom (reference 16/YH/0162) and informed written consent was obtained from all participants.

### Participants and recruitment

We recruited ten healthy adults who had no prior injuries or pathology affecting the brachial plexus. All volunteers were colleagues working within the institutions of the authors. They were recruited via posters, email circulars or word-of-mouth. All persons were assumed to have in-continuity roots.

### Image Acquisition

DTI was acquired at a field strength of 3 T (T) using a Siemens Magnetom Prisma (Siemens Healthcare Limited, Erlangen, Germany). A 64-channel head and neck coil in combination with posterior spin coils were used. Single-shot echo-planar imaging (ssEPI) was used: TR 4300 ms, TE 66 ms, in-plane resolution 2.5 mm^2^ and slice thickness 2.5 mm, field-of-view 305 × 305 × 105 mm from the C3 to T2 bodies (so as to include the origins of the rootlets for the 5–8th cervical and 1st thoracic spinal nerve roots), axial/transverse orientation, 16 interleaved non-diffusion weighted images, a b-value of 1000 s/mm^2^, 64 bipolar diffusion directions (using twice refocused spin echo) to eliminate eddy current distortion, number of signal averages 4, TrueForm b1 shim, echo spacing 0.5 ms, echo train length 445 ms, GRAPPA factor 2, receiver bandwidth 2276 Hz/px, 1st order motion correction and strong fat saturation. Two full diffusion-weighted datasets were acquired with opposing phase encoding directions; anterior–posterior and posterior-anterior. The acquisition time was 24 min per phase encoding direction.

### Pre-processing

Data were exported and pre-processed in DSI Studio (RGW; 5 years of DTI experience) to correct for susceptibility artefacts using the full diffusion-weighted datasets with opposing phase encoding directions.

### Tractography

We used DSI Studio for tractography given its superior performance in generating valid tracts^11^. The diffusion data were reconstructed by DTI using a deterministic fiber tracking algorithm^12^. The FA thresholding started at 0.1 and was reduced in decrements of 0.01 down to 0.04. At an FA threshold of 0.04 no new tracts representing the roots were propagated in any subject at any spinal level. Therefore, the FA thresholds studied were 0.04, 0.05, 0.06, 0.07, 0.08, 0.09 and 0.10, which is in keeping with other studies. The angular threshold was 70 degrees which is justified by cadaveric studies of the geometry of the roots^13^. Tracts were propagated from a single seeding region covering the entire spinal cord. The spinal rootlets and subsequent roots of the brachial plexus traverse approximately 3 cm from the cephalad longitudinal axis of the spinal cord to the exit foramen^14^ where they have a mean cross-sectional area 9 mm^2^ (ranging from 5.6 mm^2^ for the C5 to 10.8 mm^2^ for C8)^15^. From the exit foramina, the mean length of the C5/6 roots to the upper trunk union is 21 mm, the C7 root is 42 mm to its division and the C8/T1 roots are 43 mm before uniting to form the lower trunk^16^. Therefore, a region of interest (ROI) measuring 5 mm^3^ (4 voxels per slices; 8 voxels total) was placed over each root ~ 3 cm lateral to the midline of the cord, lateral to the exit foramina on FA maps (Supplementary Figs. 1, 2 and 3). Subvoxel seeding was used with a step size of ≤ 1.25 mm. A total of 250 tracts were propagated per root. Tracts shorter than 30 mm were discarded. Topology-informed pruning^17^ was applied with 10 iterations. Tract-related DTI metrics were extracted from all 10 spinal nerve roots individually. This process was performed by RGW (5 years of DTI experience) and repeated in full by a 2^nd^ independent author (GA; 6 years of DTI experience) to test the inter-rater reliability. Tracts representing the spinal nerve roots were considered valid if they originated cranially within the spinal cord and terminated beyond the ROI which covered the root (Supplementary Fig. 4). Furthermore, as regions were set to the “ROI” function within DSI Studio, tracts had to originate within the seeding region of the spinal cord, enter and exit the region of interest. This approach reduced the probability of rendering short (potentially false) tracts which terminated within the ROI.

**Figure 1:**
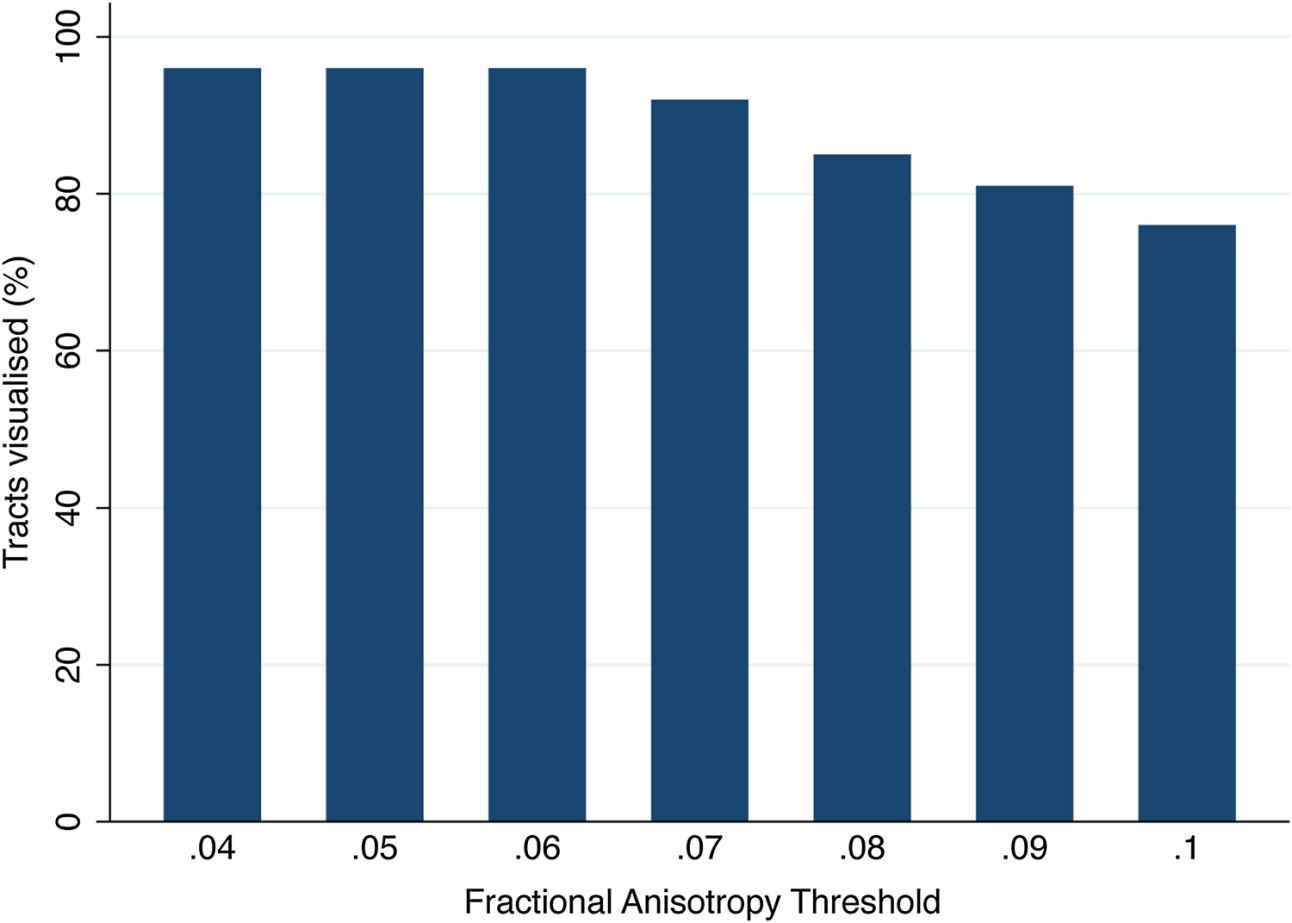
The overall proportion of visualised tracts representing roots of the brachial plexus at different FA thresholds.

**Figure 2:**
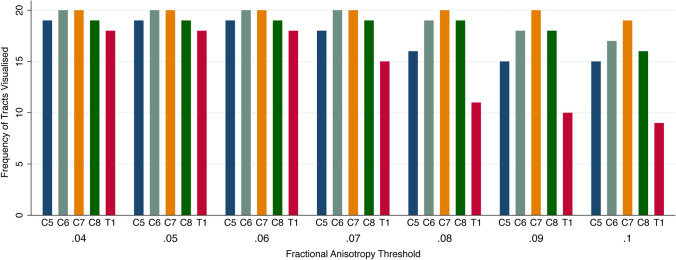
The proportion of tracts visualised representing specific roots of the brachial plexus at different FA thresholds.

**Figure 3:**
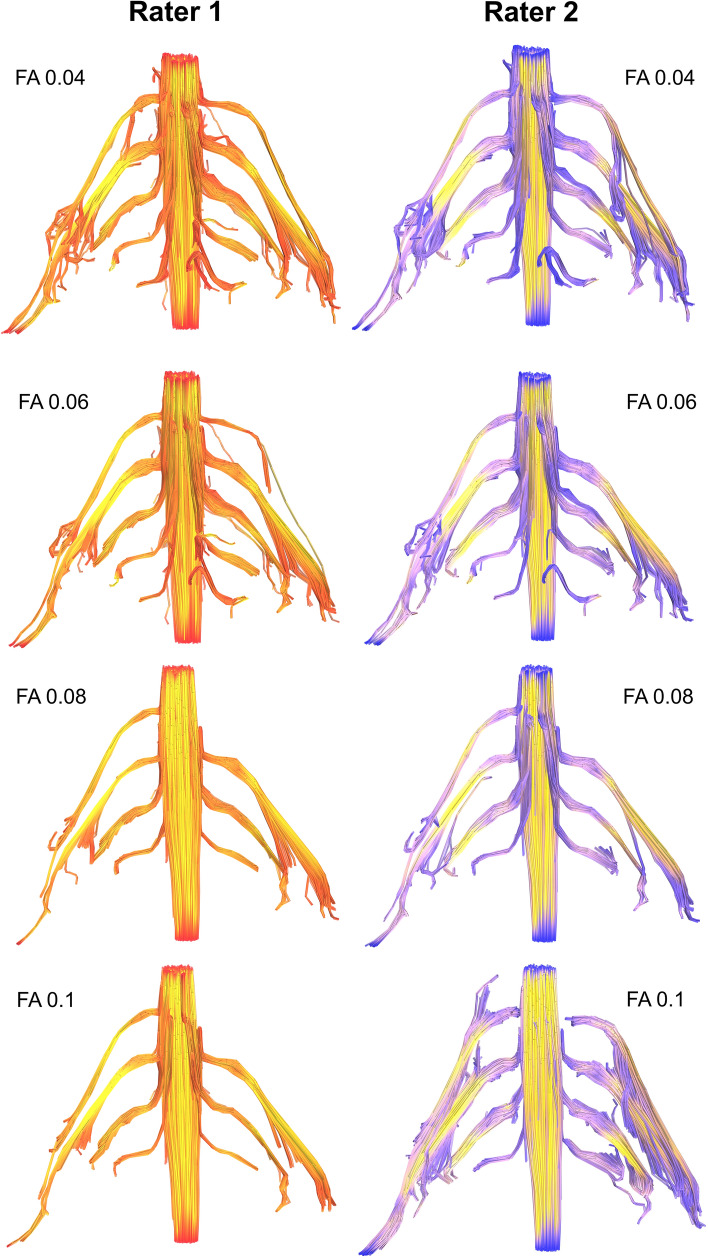
Deterministic tractography of the roots of the brachial plexus at different FA thresholds, from independent raters. The colour of the tract is determined by the local FA whereby yellow denotes a high FA (0.5), scaled to red or blue (for rater 1 or 2, respectively) which denotes low FA (0).

**Figure 4:**
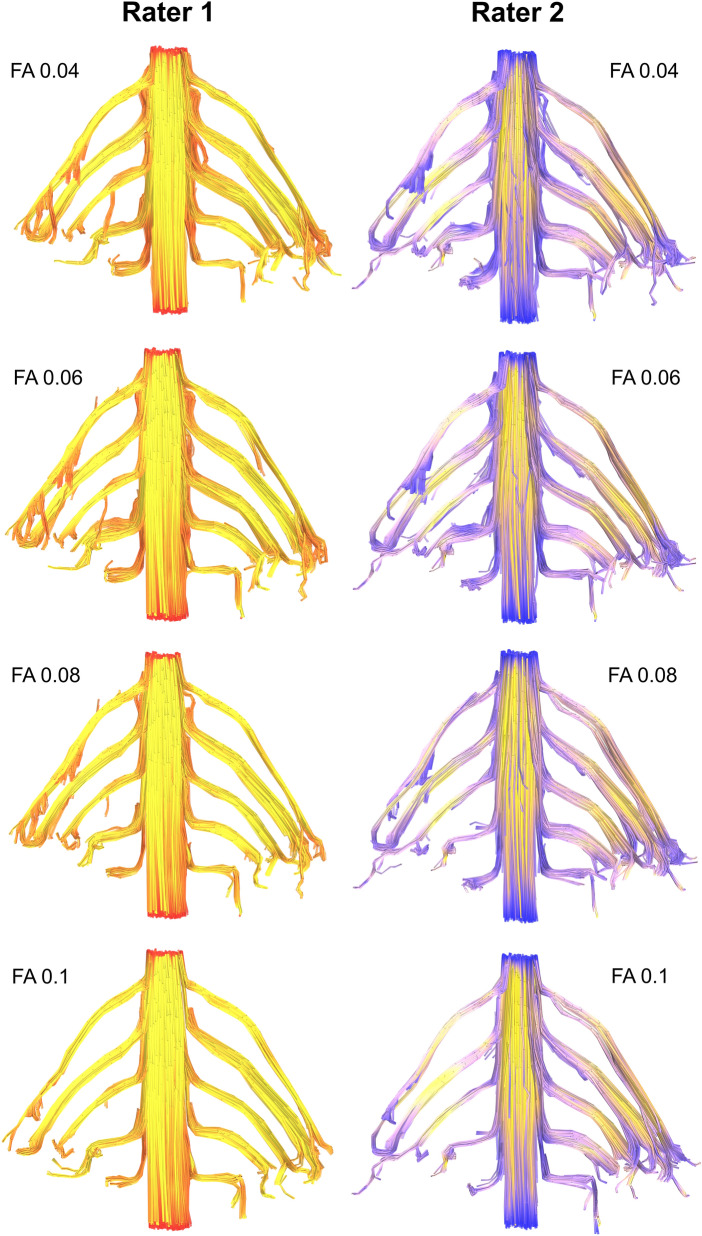
Deterministic tractography of the roots of the brachial plexus at different FA thresholds, from independent raters. The colour of the tract is determined by the local FA whereby yellow denotes a high FA (0.5), scaled to red or blue (for rater 1 or 2, respectively) which denotes low FA (0).

### Analysis

Data were analysed using Stata v15 (StataCop LLC, Texas). Age is skewed so represented by the median and interquartile range (IQR) and compared using rank methods. Other scaled variables approximated the normal so are represented by the mean (and standard deviation, SD). DTI metrics were estimated using mixed-effects linear regression. Sex, side, hand dominance and root level were the fixed effects. As measurements from ten roots within the same individual are correlated, random-effects varied by the individual. The cluster level variance and covariance parameters were estimated using the restricted maximum likelihood. Agreement was assessed using Cohen’s kappa for binary events (tract presence). To estimate the agreement (intraclass correlation coefficient, ICC) between raters’ measurements of DTI metrics, this factor added as a binary fixed-effect to the aforementioned model. The variation between raters’ extraction of FA was summarised in a Bland Altman plot.

### Ethical approval

Approval was gained from the National Research and Ethics Service of the United Kingdom (reference 16/YH/0162).

## Results

There were eight males and two females. The mean age was 28 years (SD 4, range 21–34). Eight were right-handed. DTI metrics from the roots are shown in Table 1.

**Table 1:** DTI metrics from all volunteers tabluated by the root.

Level	Side	Mean (SD) DTI values derived from propagated tracts			
		Fractional anisotropy	Mean diffusivity (× 10^–3^ mm^2^/s)	Axial diffusivity (× 10^–3^ mm^2^/s)	Radial diffusivity (× 10^–3^ mm^2^/s)
C5	Right	0.24 (0.07)	1.62 (0.58)	2.04 (0.64)	1.40 (0.56)
	Left	0.25 (0.07)	1.61 (0.59)	2.04 (0.65)	1.40 (0.57)
C6	Right	0.26 (0.08)	1.70 (0.61)	2.18 (0.69)	1.45 (0.58)
	Left	0.26 (0.08)	1.69 (0.60)	2.16 (0.68)	1.45 (0.58)
C7	Right	0.26 (0.09)	1.81 (0.57)	2.33 (0.67)	1.55 (0.54)
	Left	0.26 (0.09)	1.68 (0.54)	2.15 (0.64)	1.45 (0.50)
C8	Right	0.26 (0.10)	1.79 (0.48)	2.30 (0.57)	1.53 (0.46)
	Left	0.26 (0.09)	1.73 (0.50)	2.23 (0.62)	1.48 (0.46)
T1	Right	0.25 (0.11)	1.84 (0.55)	2.33 (0.60)	1.60 (0.56)
	Left	0.23 (0.09)	1.88 (0.55)	2.35 (0.63)	1.66 (0.53)
All roots	Right	0.26 (0.09)	1.74 (0.56)	2.23 (0.64)	1.50 (0.54)
	Left	0.25 (0.08)	1.72 (0.55)	2.19 (0.64)	1.48 (0.52)
Overall		0.25 (0.09)	1.73 (0.56)	2.21 (0.64)	1.49 (0.53)

### Variability in DTI metrics between subjects

There was significant variability between subjects DTI metrics (Table 2). The mean FA varied between subjects by 2% (95% CI 1%, 3%); ICC 0.16, *p* < 0.001). The mean MD varied by a mean of 0.15 mm^2^/s × 10^–3^ (95% CI 0.09, 0.24); ICC 0.40, *p* < 0.001). The mean AD varied by a mean of 0.18 mm^2^/s × 10^–3^ (95% CI 0.11, 0.29); ICC 0.37, *p* < 0.001). The RD varied by a mean of 0.14 mm^2^/s × 10^–3^ (95% CI 0.09, 0.22); ICC 0.40, *p* < 0.001).

**Table 2:** DTI metrics from all roots the brachial plexus tabulated by individual.

Volunteer	Side	Mean (SD) DTI values derived from propagated tracts			
		Fractional anisotropy	Mean diffusivity (× 10^–3^ mm^2^/s)	Axial diffusivity (× 10^–3^ mm^2^/s)	Radial diffusivity (× 10^–3^ mm^2^/s)
1	Right	0.27 (0.09)	1.88 (0.65)	2.42 (0.72)	1.60 (0.63)
	Left	0.27 (0.09)	1.85 (0.65)	2.39 (0.75)	1.57 (0.62)
2	Right	0.29 (0.10)	1.83 (0.58)	2.40 (0.69)	1.54 (0.55)
	Left	0.29 (0.10)	1.77 (0.57)	2.33 (0.67)	1.50 (0.54)
3	Right	0.23 (0.09)	1.90 (0.62)	2.41 (0.71)	1.64 (0.60)
	Left	0.24 (0.08)	1.86 (0.61)	2.32 (0.66)	1.64 (0.59)
4	Right	0.26 (0.09)	1.78 (0.56)	2.26 (0.59)	1.54 (0.56)
	Left	0.25 (0.09)	1.78 (0.55)	2.27 (0.60)	1.54 (0.54)
5	Right	0.31 (0.11)	1.71 (0.46)	2.31 (0.55)	1.41 (0.45)
	Left	0.27 (0.08)	1.67 (0.47)	2.17 (0.56)	1.42 (0.45)
6	Right	0.28 (0.10)	1.50 (0.51)	1.96 (0.57)	1.28 (0.5)
	Left	0.26 (0.09)	1.49 (0.46)	1.90 (0.51)	1.29 (0.45)
7	Right	0.27 (0.09)	1.79 (0.53)	2.30 (0.62)	1.52 (0.50)
	Left	0.26 (0.09)	1.87 (0.47)	2.41 (0.68)	1.61 (0.57)
8	Right	0.29 (0.09)	1.54 (0.57)	2.02 (0.69)	1.29 (0.52)
	Left	0.29 (0.09)	1.48 (0.47)	1.96 (0.57)	1.25 (0.43)
9	Right	0.25 (0.09)	1.90 (0.59)	2.41 (0.64)	1.65 (0.60)
	Left	0.27 (0.09)	1.99 (0.63)	2.56 (0.72)	1.70 (0.60)
10	Right	0.24 (0.09)	1.80 (0.51)	2.26 (0.56)	1.57 (0.50)
	Left	0.24 (0.09)	1.77 (0.55)	2.23 (0.62)	1.55 (0.53)

### Variability in DTI metrics between sex, side, and handedness

The FA was statistically higher on the right side, although the absolute difference was very small (mean difference 0.01 [95% CI 0.002, 0.02]). Similarly, the FA was statistically higher in the roots of the dominant limb (mean difference 0.01 [95% CI 0.01, 0.2]) although the absolute difference was again very small. There was no significant interaction between hand dominance and side (*p* = 0.05). There was no statistically significant difference in the FA measurements from the roots of men and women (mean difference 0.01 [95% CI − 0.02, 0.04]).

The MD was statistically higher on the right side (mean increase 0.02 × 10^–3^ mm^2^/s [95% CI 0.002, 0.04]) and in the roots of the non-dominant limb (mean increase 0.02 × 10^–3^ mm^2^/s [95% CI 0.005, 0.04]), although the absolute differences were very small. There was no significant interaction between hand dominance and side (p = 0.05). There was no statistically significant difference in the MD of the roots between men and women (mean difference 0.08 × 10^–3^ mm^2^/s [95% CI − 0.16, 0.32]).

The AD was statistically higher on the right side (mean increase 0.05 × 10^–3^ mm^2^/s [95% CI 0.03, 0.07]) and in the non-dominant limb (mean increase 0.05 × 10^–3^ mm^2^/s [95% CI 0.03, 0.08]), although the absolute differences were very small. There was no significant interaction between hand dominance and side (*p* = 0.05). Again, there was no statistically significant difference in the AD of the roots between men and women (mean difference 0.07 × 10^–3^ mm^2^/s [95% CI − 0.22, 0.36]).

There was no statistically significant difference in the RD between the right and left side (mean difference 0.003 × 10^–3^ mm^2^/s [95% CI − 0.01, 0.02]), the dominant limb and non-dominant limbs (mean difference 0.008 × 10^–3^ mm^2^/s [95% CI − 0.008, 0.03]), or men and women (mean difference 0.08 × 10^–3^ mm^2^/s [95% CI -0.13, 0.31]).

### FA thresholds: tractography

**Figure 5:**
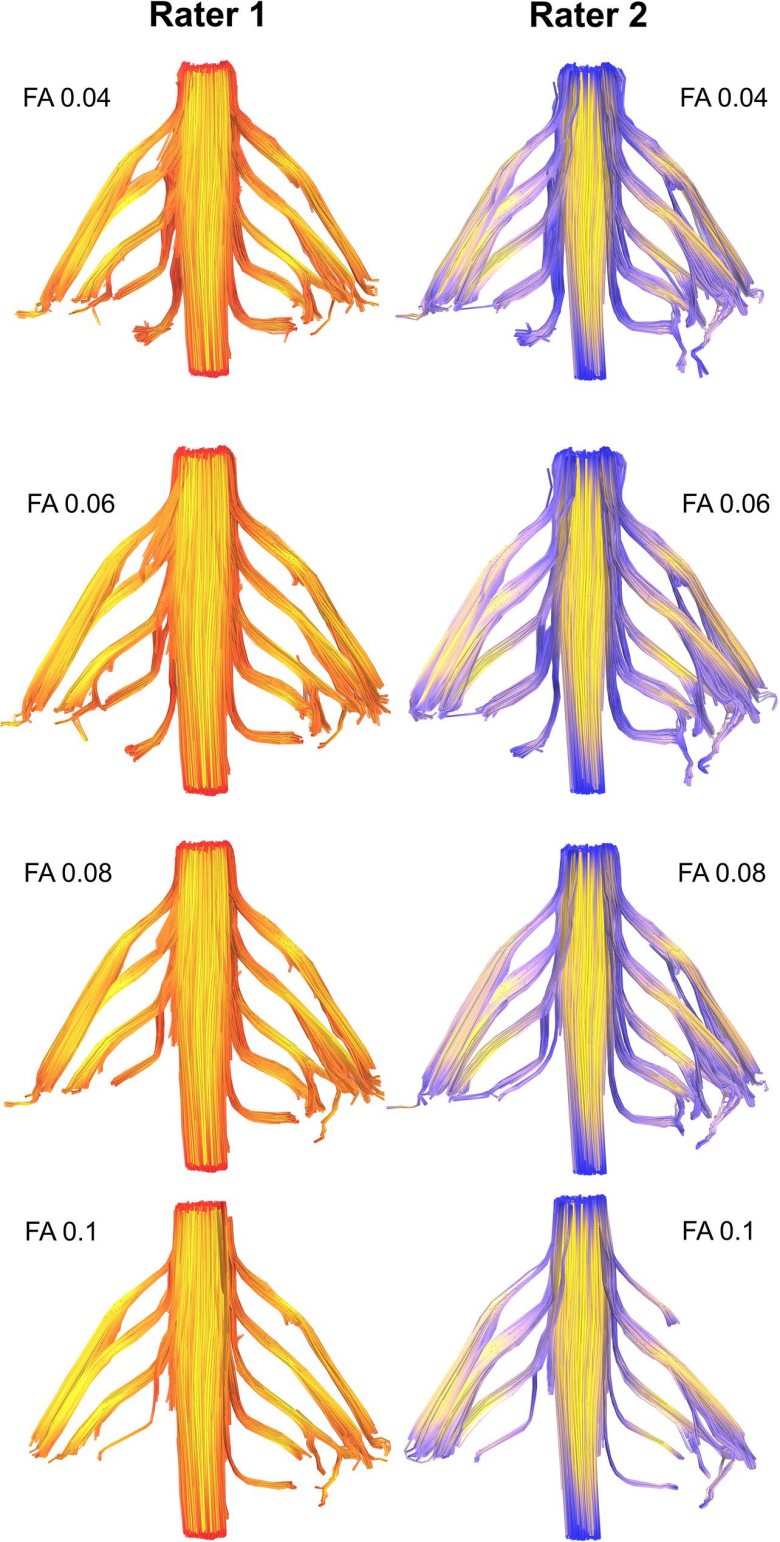
Deterministic tractography of the roots of the brachial plexus at different FA thresholds, from independent raters. The colour of the tract is determined by the local FA whereby yellow denotes a high FA (0.5), scaled to red or blue (for rater 1 or 2, respectively) which denotes low FA (0).

Overall, at higher FA thresholds fewer tracts were propagated (Fig. 1 and Supplementary Table 1); this was principally due to the failure to render tracts representing the T1 root (Fig. 2) and to a lesser extent the other cervical roots (Figs. 3, 4, 5).

There was no statistically significant difference in the proportion of tracts rendered at FA thresholds of 0.04, 0.05 or 0.06. Overall, at FA thresholds of ≤ 0.06 tracts representing the roots were propagated for 96% of spinal levels (Figs. 3, 4, 5 and Supplementary Table 1). In comparison, thresholding the FA at 0.07 yield 4% fewer tracts (*p* = 0.2), 0.08 yield 11% fewer tracts (*p* = 0.008), 0.09 yield 15% fewer tracts (*p* = 0.001) and 0.1 yield 20% fewer tracts (*p* < 0.001). This appears to be due to partial volume effects (reduce by cerebrospinal fluid) as the FA in the rootlets is substantially lower than the spinal cord and extraforaminal portions of the spinal roots (Figs. 3, 4, 5).

### FA thresholds: tract length

**Figure 6:**
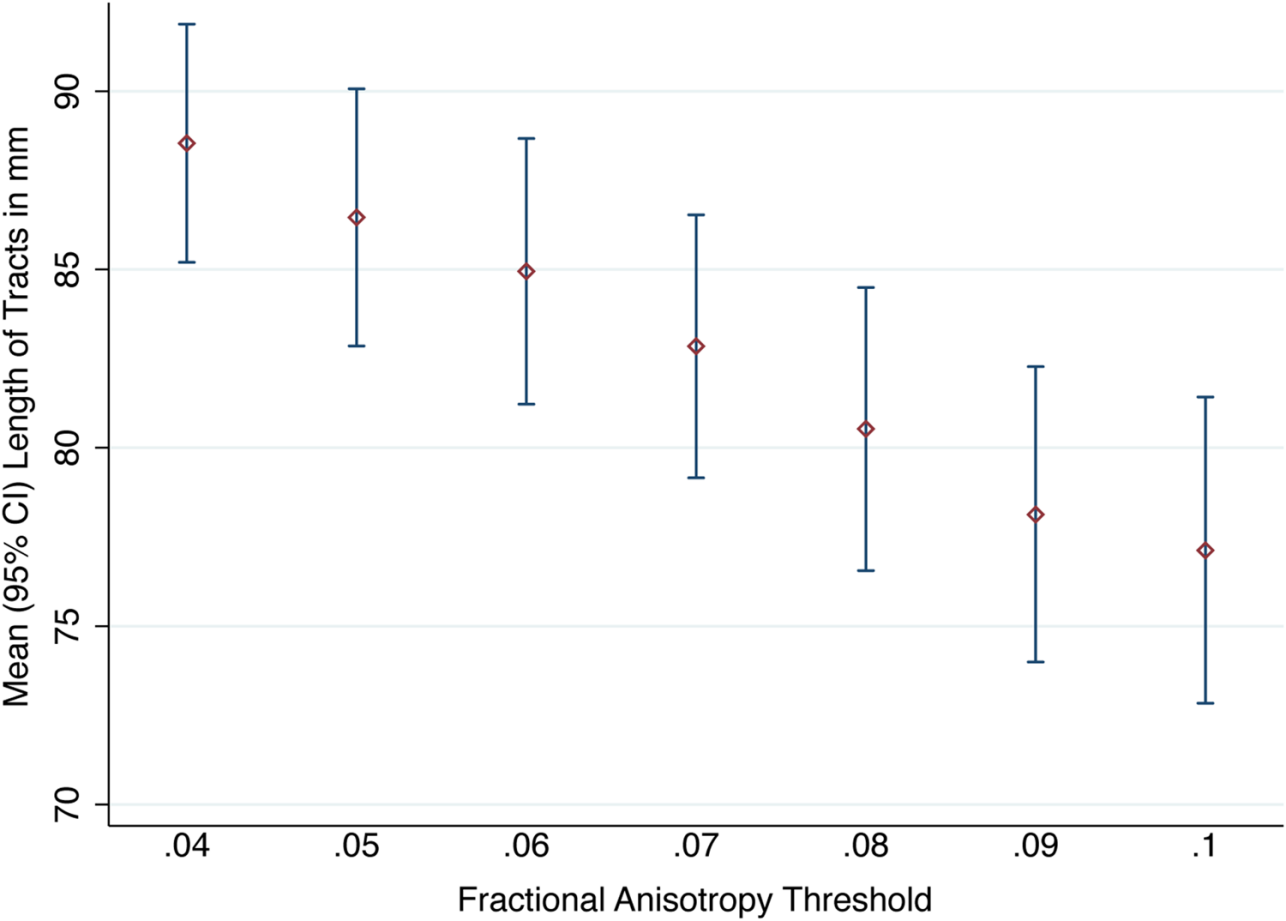
The mean length of tracts (in mm) by FA threshold.

Overall, the FA threshold was strongly associated with tract length (Fig. 6), whereby for every unit increase in the FA threshold, propagated tracts were 2 mm shorter (95% CI 1.3, 2.7).

Using a FA threshold of 0.06, there was no significant difference in the length of tracts between the left and right side (mean difference 1.37 mm [95% CI − 3.22, 6.00]), the dominant and non-dominant limb (mean difference 2.24 mm [95% CI − 2.00, 6.47]); or males and females (mean difference 5.30 mm [95% CI − 0.10, 10.7]).

### Inter-rater agreement

There was 99% agreement between raters’ assessment of the presence of a tract representing the roots at different FA thresholds (Cohen’s kappa 0.92, *p* < 0.001). There was strong agreement between raters’ FA measurements from the roots (Supplementary Fig. 5) with < 0.1% variability (mean difference 0.008 [95% CI − 0.004, 0.01]; ICC 0.001). There was strong agreement between raters’ assessment of MD (mean difference 0.02 [95% CI − 1.7, 0.4]; ICC 0.016), AD (mean difference 0.01 [95% CI -2.1, 0.5]; ICC 0.02) and RD (mean difference 0.06 [95% CI − 1.5, 0.4]; ICC 0.01).

## Discussion

This work helps to define the FA thresholds needed for tractography of the roots of the brachial plexus in healthy adults. This information may be used to inform decisions regarding tractography conditions in future studies of the brachial plexus in adults.

We have shown that the DTI metrics and FA thresholds for tractography of the brachial plexus appear to be different to the white matter tracts in the brain. Lebel and colleagues^18^ showed that the mean FA of white matter tracts in the adult brain is 0.36 to 0.54, depending on the structure. However, we have shown that the extraforaminal roots have a lower mean FA. Whilst the brachial plexus roots are akin to white central matter tracts (in that they are myelinated and highly ordered), the fascicular arrangement in the roots is substantially different in two main ways:

1. The density of axons (per mm^2^) in the roots of the brachial plexus is 5 times less than the genu of the corpus callosum^19^ and half that of the pyramidal tracts of the spinal cord^20^. In the corpus callosum there are approximately 38,000 myelinated fibres per mm^2^ (70% are < 1** μm** in diameter and exhibit highly restricted diffusion). In the spinal cord there are 10–20,000 axons per mm^2^ (depending on the spinal level) and 52% are < 1** μm** in diameter^21^. The roots of the brachial plexus have a variable axon density, specifically 6750 per mm^2^ in C5, 8448 per mm^2^ in C6, 8665 per mm^2^ in C7, 5708 per mm^2^ in C8 and ~10,000 per mm^2^ in T1^15,22^. Compared to the central nervous system, the fourfold lower density of axons per mm^2^ partly explains the lower FA.
2. Numerous histological studies have shown that the topography of the nerves of the brachial plexus changes every few millimetres and there is substantial fascicular sharing/cross-over^23,24^. Unlike the central white matter tracts, axons and fascicles in the roots bifurcate, merge, weave and exchange throughout the brachial plexus from the level of the intradural rootlets to the target organ^25^. These intraneural and interfascicular connections will increase signal dispersion and may further explain why the FA is lower in the roots of the brachial plexus than in central white matter tracts.

Although our findings are in agreement with the metrics reported in many DTI studies of the brachial plexus^4–7,9,10,26^ (Supplementary Table 2), our FA values are slightly lower. FA is a function of numerous factors such as the b-value and number of diffusion encoding directions^27,28^. Therefore, it is plausible that other studies (of all which use smaller b-values and fewer directions) may be reporting upwardly biased estimates of the FA which underestimate dispersion^29,30^. Partial volume effects may also contribute to the lower FA because we sampled data at a lower resolution than Ho^10^, for example, meaning that data from the rootlets (which are bathed in cerebrospinal fluid) may be subject to more partial volume effects as shown by the red/blue portions of Figs. 3, 4 and 5. Future studies should consider acquiring data from higher b-values (which are sensitive to restricted diffusion) and explore how different models (such as NODDI) or model-free methods (such as generalised q-space imaging) effect the diffusion metrics.

As with different regions of the brain, the ideal tractography conditions for peripheral nerves differ from region-to-region. Therefore, it is important that researchers and clinicians have evidence on which to base their selection of brachial plexus tracking thresholds so as to propagate tracts which represent real connections and equally, avoid propagating false tracts, e.g. into skeletal muscle which has a similar and positively correlated FA^31^. The typical FA thresholds used for tractography in the brain is ≥ 0.1, but this is too high for the roots of the brachial plexus because the microstructure is different and thus, tracts representing real connections would not propagate^5^. Similar studies of FA thresholding in the brain have shown that it has considerable effect on the number, density and directionality of tracts, as well as tract-based estimates of anisotropy and diffusivity^32–34^. Furthermore, one study showed that with conventional FA thresholds (of ≥ 0.1), modern tracking algorithms which are robust to crossing-fibres missed clinically-important tracts in the brain, whereas real-time threshold reductions to a FA of 0.06 yielded more meaningful and clinically-useful tractograms^34^. Therefore, we argue that using a FA threshold generalised from tractography studies in the brain or elsewhere is inappropriate.

### Limitations

The VoTEM, TraCED and ISMRM Tractography challenges^11,35,36^, and numerous phantom studies^37–39^, have shown that DTI is reproducible across scanners and pulse sequences^40^. Similarly, DTI provides reconstructions which are closest to the ground truth, with the greatest valid:non-valid connection ratio^41^. However, the prevalence of false connections remains high and the utility of tractograms still remains unclear^42^. Whilst we used topology-informed pruning^17^ to remove false tracts, this technology has not be validated in peripheral nerves. Equally, we have shown that altering the FA threshold affects tract length, with some tracts potentially being falsely propagated or inappropriately long. Therefore, our findings should be interpreted with caution and we recognise the need for further studies examining the association between tractograms (generated under different conditions) and the ground truth of anatomical dissections.

The literature is lacking reliable research concerning DTI metrics in human peripheral nerves in health, after injury or disease onset, and how these relate to nerve degeneration and regeneration. Until these fundamental questions are answered, the translational value of our work can only be speculated. Equally, this study considers data from the first 5–7 cm of the brachial plexus and our findings should not be extrapolated beyond this anatomical area. We advocate the development of a biobank for diffusion MRI of peripheral nerves; ideally this would contain data from healthy adults, recently deceased donors and fixed cadavers to permit comprehensive analysis of the effects of numerous co-variables on DTI metrics throughout the length of the nerves.

The 1^st^ thoracic root was the least frequently reconstructed tract perhaps due to: low signal-to-noise given its distance from the coil, residual/uncorrected eddy-current and/or susceptibility artefacts from the lung apex and adjacent 1^st^ rib, flow and partial volume effects due to the neighbouring great vessels, and spatial mis-mapping from apical breathing. Future work should compare different software for artefact correction and approaches (forward full dataset with reversed b0s vs. full opposing polarity datasets) with the aim of defining the ideal pipeline.

Our DTI protocol is long (to optimise SNR) which may not be tolerable for patients. Our sample is small and originates from a single scanner within one centre, which may limit the generalisability. In an effort to reduce scan time and thus, the opportunity to scan more people to generate estimates of normality, our future work will explore different q-space sampling strategies, signal averaging, multiband (simultaneous multislice) imaging and pre-processing pipelines.

We observed differences in the FA and MD of right versus left sided roots, which was independent of handedness. This is difficult to explain biologically, so may represent: (a) type 1 errors, (b) a insufficient data from left-handed individuals which might translate to less precise estimates of the variance, (c) idiosyncrasy related to the study design or sample, (d) field inhomogeneities or otherwise. Further work (and ideally an individual participant data meta-analysis, using data from a biobank) could investigate how DTI metrics from each root differ in the dominant versus non-dominant limb.

## Conclusions

The fractional anisotropy threshold required to generate tractograms of the roots of the brachial plexus appears to be lower than those used in the white matter pathways in the brain. We provide estimates of the probability of generating true tracts for each spinal nerve root of the brachial plexus, at different fractional anisotropy thresholds and show how this affects tract-related metrics.

## Supplementary Information

Supplementary Information.
