# Supplementary material for: Fractional anisotropy thresholding for deterministic tractography of the roots of the brachial plexus
Source: Sci Rep. 2021 Jan 8;11:80. doi: 10.1038/s41598-020-79840-8 (PMC7794285; doi:10.1038/s41598-020-79840-8)
Supplement: Supplementary file 1 — Supplementary Information. [file 41598_2020_79840_MOESM1_ESM.docx]

**Title**

Fractional Anisotropy Thresholding for Deterministic Tractography of the Roots of the Brachial Plexus using Diffusion Tensor Imaging

**Authors**

Ryckie G Wade MBBS MSc MClinEd MRCS FHEA

Irvin Teh BE MBiomedE PhD

Gustav Andersson MD PhD

Fang-Cheng Yeh MD PhD

Mikael Wiberg MD PhD

Grainne Bourke MB BCh BAO FRCSI FRCS(Plast)

**Supplementary Figure 1.** FA maps from a single volunteer. The top image is an axial-oblique slice showing the 7^th^ cervical root arising from the spinal cord (red arrow) transitioning into the middle cord (yellow arrow) laterally. The lower image is a coronal-oblique slice showing the spinal cord (again, red arrow) as well as the left 5^th^ and 6^th^ cervical roots (green and blue arrow, respectively).


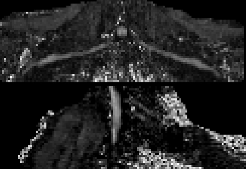


**Supplementary Figure 2.** The same axial-oblique FA map shown in Supplementary Figure 1 with the regions of interest for the spinal cord (red), left C7 root (yellow) and right C7 root (pink).


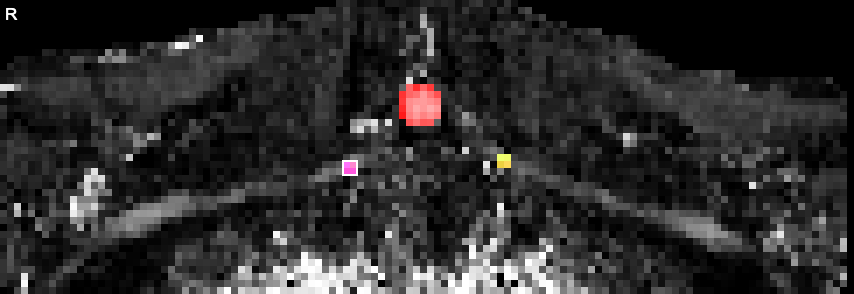


**Supplementary Figure 3.** A customised tractograms with regions of interest overlaid. Each root was tracked individually. All tracts were seeded from within the region of interest covering the spinal cord. Tracts representing each root were generated individually; tracts were required to originate in the seeding region and pass through (enter and exit) a spherical region of interest overlying the root.


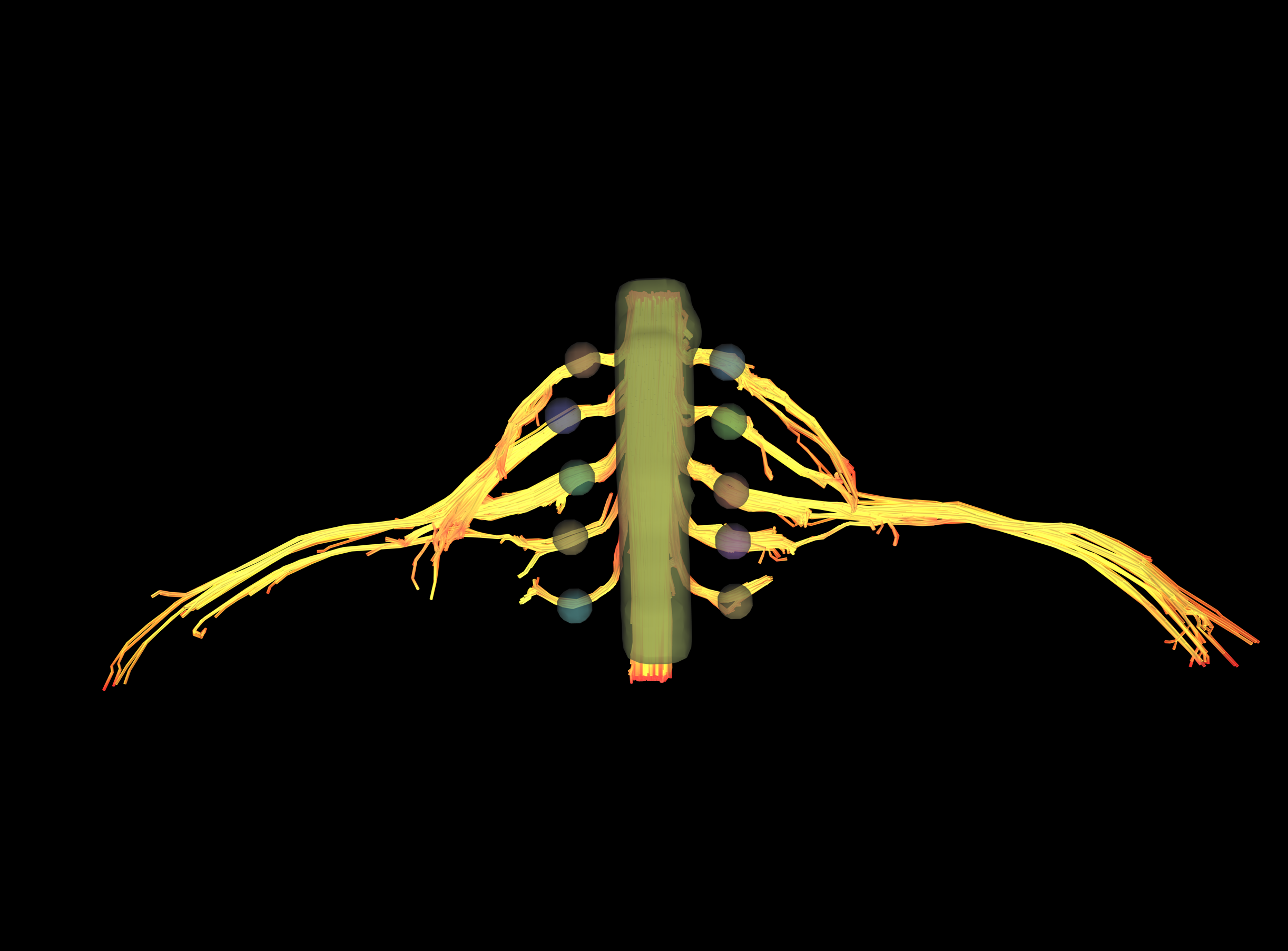


**Supplementary Figure 4.** A tractogram from one volunteer with regions of interest overload. Tracts coloured yellow were defined as “valid” representations of the roots. The left T1 (red) tract-bundle was considered valid despite the caudally originating (false) tracts. Both the dark and light blue coloured tracts representing the right C8 and right T1 roots were considered “invalid” (by both raters) because tract-bundles originated exclusively from caudally within the spinal cord and there were numerous false, looping tracts which do not resemblance to the typical morphology of the 1^st^ thoracic root.


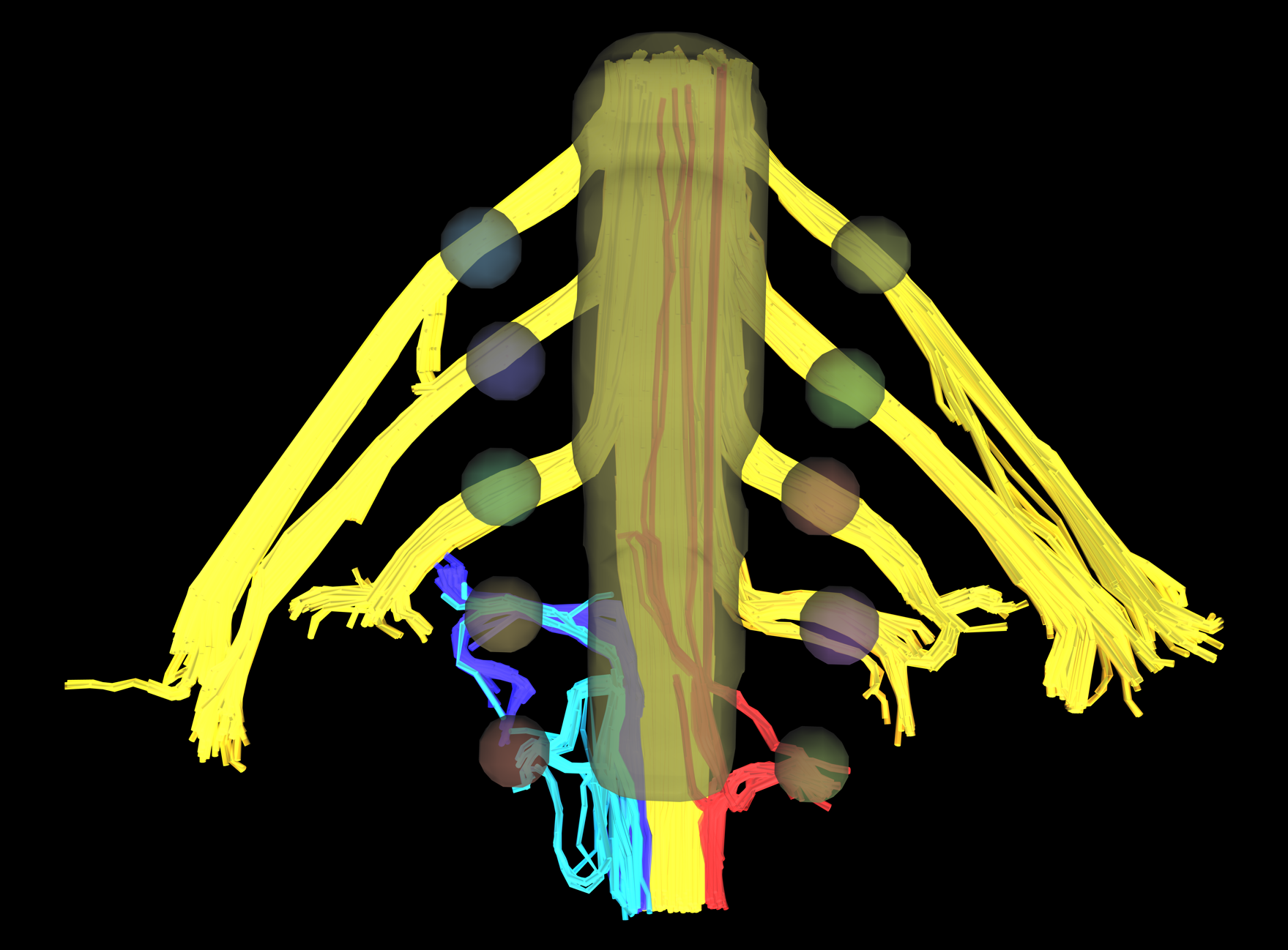


**Supplementary Figure 5.** Bland Altman Plot showing strong agreement in the assessment of the fractional anisotropy of the roots between raters.

**
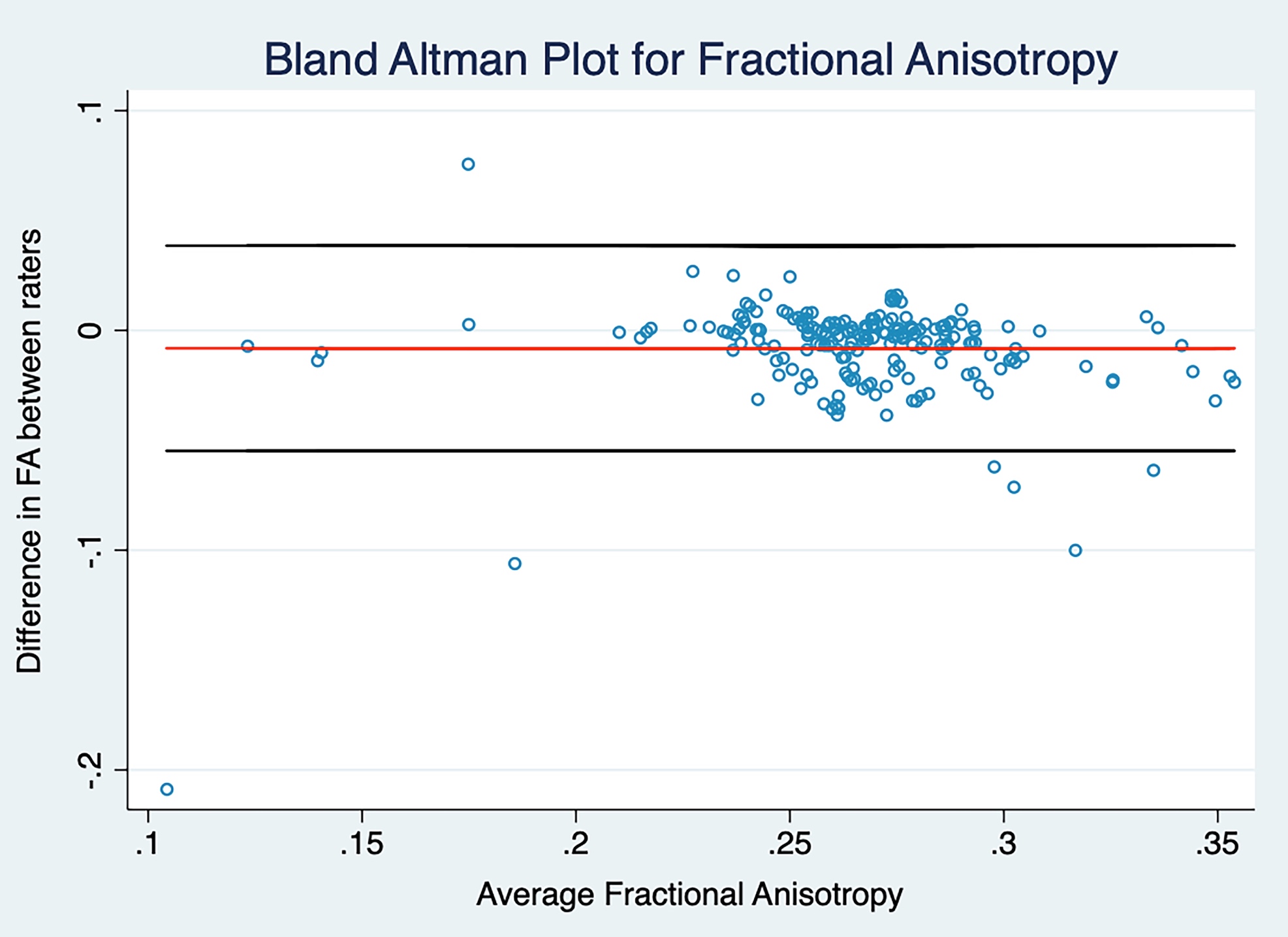
**

**Supplementary Table 1.** Core features of the pulse sequence and metrics for studies investigating the roots brachial plexus using DTI

| **Study** | **Fields Strength** | **Sequence** | **ROI position** | **ROI size/ volume** | **FA** | **MD (mm^2^/s x10^-3^)** |
| --- | --- | --- | --- | --- | --- | --- |
| Current study | 3T | ssEPI: b0/1000 s/mm^2^, 64 directions, 8 averages (4 per phase encoding direction), TE 66ms, TR 4300ms, 2.5mm isotropic. | Extraforaminal roots, 3cm lateral to the midline of the spinal cord | 5mm^3^  (8 voxels) | Mean 0.25 (SD) | Mean 1.73  (SD 0.56) |
| Wade (2020) | 3T | ssEPI: b0,1000 s/mm^2^, 20 directions, 4 averages, TE 66ms, TR 4300ms, 2.5mm isotropic. | Lateral recess of the intervertebral foramina | 10mm^2^ | Mean 0.28  (SD 0.08) | Mean 1.79  (SD 0.18) |
| Tagliafico (2011) | 3T | ssEPI: b0/1000s/mm^2^, 32 directions, averaging not described, TE “minimum”, TR 16675ms, 2mm slice thickness. | Not described | 2mm^2^ | 0.27 to 0.43 | 1.41 to 1.81 |
| Ho (2017) | 3T | ssEPI: b0/800s/mm^2^, 30 directions, single acquisition, TE 67ms, TR 5600ms, 1.9mm isotropic. | Distal to the ganglia | 11m^2^ | 0.39 to 0.44 | 1.49 to 1.62 |
| Ho (2019) | 3T | rsEPI: b0/900s/mm^2^, 4 averages, TE 57ms, TR 3030ms, 2.4mm isotropic, *number of directions not described.*  ssEP: b0/900s/mm^2^, 4 averages, TE 59ms, TR 5800ms, 2.4mm isotropic, *number of directions not described.* | Distal to the ganglia | Bespoke | Mean 0.37  (SD 0.02) | Not reported |
| Oudeman (2018) | 3T | ssEPI: b0/800s/mm^2^, 15 directions, TE 77ms, TR 5969ms, 6 averages, 3mm isotropic. | Close to the ganglia | Not described | Mean 0.33  (SD 0.04) | Mean 1.36  (SD 0.13) |
| Vargas (2010) | 1.5T | ssEPI: b0/900 s/mm^2^), 30 directions, no repetitions, TE 78ms, TR 9000ms, 2mm isotropic voxels | “Several places on the spinal cord, trunks and cords” | 2mm^2^ | Mean 0.30 | Mean 1.70 |
| Su (2019) | 3T | rsEPI: 0/900 s/mm^2^, 20 directions, 4 averages, TE 92ms, TR 6000ms, 2x2x3mm voxels | Postganglionic roots | Not described | 0.40 to 0.48 | 1.39 to 1.54 |

**Supplementary Table 2.** DTI metrics by the FA threshold used for tractography and root

| **FA threshold** | **Level** | **Tract properties** | | |
| --- | --- | --- | --- | --- |
|  |  | **Tracts visualised (%)** | **Length (mm)** | **Volume (tracts mm^-3^)** |
| 0.04 | C5 | 19 (95) | 75 (18) | 3192 (903) |
|  | C6 | 20 (100) | 86 (17) | 3582 (868) |
|  | C7 | 20 (100) | 88 (17) | 3581 (868) |
|  | C8 | 19 (95) | 84 (16) | 3234 (803) |
|  | T1 | 18 (90) | 84 (21) | 2893 (807) |
|  | **Overall** | **96 (96)** | **87 (3)** | **3410 (179)** |
| 0.05 | C5 | 19 (95) | 73 (17) | 2044 (875) |
|  | C6 | 20 (100) | 85 (17) | 2501 (896) |
|  | C7 | 20 (100) | 88 (16) | 3429 (756) |
|  | C8 | 19 (95) | 84 (16) | 3071 (769) |
|  | T1 | 18 (90) | 80 (23) | 2711 (781) |
|  | **Overall** | **96 (96)** | **84 (4)** | **3198 (193)** |
| 0.06 | C5 | 19 (95) | 73 (17) | 3029 (920) |
|  | C6 | 20 (100) | 85 (17) | 3392 (914) |
|  | C7 | 19 (95) | 87 (16) | 3353 (774) |
|  | C8 | 19 (95) | 84 (16) | 2937 (813) |
|  | T1 | 18 (90) | 79 (24) | 2510 (875) |
|  | **Overall** | **96 (96)** | **81 (4)** | **3002 (208)** |
| 0.07 | C5 | 18 (90) | 71 (17) | 2903 (919) |
|  | C6 | 20 (100) | 84 (17) | 3196 (917) |
|  | C7 | 20 (100) | 86 (17) | 3188 (731) |
|  | C8 | 19 (95) | 84 (17) | 2792 (758) |
|  | T1 | 15 (75) | 75 (26) | 2199 (840) |
|  | **Overall** | **92 (92**) | **81 (4)** | **2903 (201)** |
| 0.08 | C5 | 16 (80) | 71 (17) | 2730 (819) |
|  | C6 | 19 (95) | 83 (18) | 3110 (903) |
|  | C7 | 20 (100) | 84 (18) | 2977 (743) |
|  | C8 | 19 (95) | 81 (18) | 2594 (772) |
|  | T1 | 11 (55) | 70 (27) | 1922 (813) |
|  | **Overall** | **85 (85)** | **81 (4)** | **2747 (224)** |
| 0.09 | C5 | 15 (75) | 69 (17) | 2587 (740) |
|  | C6 | 18 (90) | 81 (17) | 2893 (916) |
|  | C7 | 20 (100) | 81 (18) | 2870 (814) |
|  | C8 | 18 (90) | 80 (19) | 2375 (705) |
|  | T1 | 10 (50) | 68 (26) | 1780 (693) |
|  | **Overall** | **81 (81)** | **80 (5)** | **2473 (230)** |
| 0.1 | C5 | 15 (75) | 67 (14) | 2264 (739) |
|  | C6 | 17 (85) | 81 (18) | 2759 (845) |
|  | C7 | 19 (95) | 81 (19) | 2582 (738) |
|  | C8 | 16 (80) | 76 (22) | 2158 (669) |
|  | T1 | 9 (45) | 72 (23) | 1643 (630) |
|  | **Overall** | **76 (76)** | **78 (5)** | **2280 (220)** |
